# Pseudorabies Virus Infected Porcine Epithelial Cell Line Generates a Diverse Set of Host MicroRNAs and a Special Cluster of Viral MicroRNAs

**DOI:** 10.1371/journal.pone.0030988

**Published:** 2012-01-23

**Authors:** Yi-Quan Wu, Di-Jun Chen, Hua-Bin He, Dong-Sheng Chen, Ling-Ling Chen, Huan-Chun Chen, Zheng-Fei Liu

**Affiliations:** 1 State Key Laboratory of Agricultural Microbiology, College of Veterinary Medicine, Huazhong Agricultural University, Wuhan, People's Republic of China; 2 Center for Bioinformatics, College of Life Science and Technology, Huazhong Agricultural University, Wuhan, People's Republic of China; 3 Key Lab of Agricultural Animal Genetics, Breeding, and Reproduction of Ministry of Education & Key Laboratory of Swine Genetics and Breeding of Ministry of Agriculture, College of Animal Science, Huazhong Agricultural University, Wuhan, People's Republic of China; Cardiovascular Research Institute Maastricht - Maastricht University, The Netherlands

## Abstract

Pseudorabies virus (PRV) belongs to *Alphaherpesvirinae* subfamily that causes huge economic loss in pig industry worldwide. It has been recently demonstrated that many herpesviruses encode microRNAs (miRNAs), which play crucial roles in viral life cycle. However, the knowledge about PRV-encoded miRNAs is still limited. Here, we report a comprehensive analysis of both viral and host miRNA expression profiles in PRV-infected porcine epithelial cell line (PK-15). Deep sequencing data showed that the ∼4.6 kb intron of the large latency transcript (LLT) functions as a primary microRNA precursor (pri-miRNA) that encodes a cluster of 11 distinct miRNAs in the PRV genome, and 209 known and 39 novel porcine miRNAs were detected. Viral miRNAs were further confirmed by stem-loop RT-PCR and northern blot analysis. Intriguingly, all of these viral miRNAs exhibited terminal heterogeneity both at the 5′ and 3′ ends. Seven miRNA genes produced mature miRNAs from both arms and two of the viral miRNA genes showed partially overlapped in their precursor regions. Unexpectedly, a terminal loop-derived small RNA with high abundance and one special miRNA offset RNA (moRNA) were processed from a same viral miRNA precursor. The polymorphisms of viral miRNAs shed light on the complexity of host miRNA-processing machinery and viral miRNA-regulatory mechanism. The swine genes and PRV genes were collected for target prediction of the viral miRNAs, revealing a complex network formed by both host and viral genes. GO enrichment analysis of host target genes suggests that PRV miRNAs are involved in complex cellular pathways including cell death, immune system process, metabolic pathway, indicating that these miRNAs play significant roles in virus-cells interaction of PRV and its hosts. Collectively, these data suggest that PRV infected epithelial cell line generates a diverse set of host miRNAs and a special cluster of viral miRNAs, which might facilitate PRV replication in cells.

## Introduction

Pseudorabies virus (PRV), the etiologic pathogen of Aujeszky's disease causing huge economic loss in pig industry, belongs to the *Alphaherpesvirinae* subfamily with a broad host range and pantropic property, and has been shown to be a useful model organism for the studies of herpesvirus biology [1], [2], [3]. PRV genome is about 142 kb long and encodes 70 proteins and a large latency transcript (LLT). PRV LLT, an 8.4 kb polyadenylated RNA, is the only abundant transcript during the latent stage and is split into different sizes including a ∼4.6 kb stable intron as in the case of HSV-1 [4], [5], [6]. Latency associated transcript (LAT) expression is involved in the latency-reactivation cycle by inhibiting apoptosis of latently infected neurons, viral gene expression, and productive infection [7].

MicroRNAs (miRNAs) are endogenous non-coding RNAs that can direct the inhibition of target mRNAs expression via complementary sites in the 3′ untranslated region (3′ UTR) either by transcriptional destabilization or translational repression [8]. Several reports revealed that miRNAs play an integral part of gene regulatory networks and are involved in various aspects of cellular pathways and processes [9]. Most miRNAs are initially transcribed by RNA polymerase II into longer transcripts called primary miRNAs (pri-miRNAs) precursor, and then pri-miRNAs are cleaved by the nuclear RNase III enzyme Drosha to liberate 60nt∼80-nt hairpin structures called pre-miRNAs. Following translocation from the nucleus to the cytoplasm, the pre-miRNAs are further cleaved by a second RNAase III, called Dicer, to liberate the miRNA duplexes. Generally, one strand of this duplex is degraded, whereas the other strand is incorporated into the RNA-induced silencing complex (RISC), where it acts as a guide RNA to direct RISC to target mRNAs [8].

In addition to numerous cellular miRNAs from plants and animals, recent studies have also confirmed the existence of viral miRNAs. Current evidences indicate that viruses harness host miRNA biogenesis system to control the expression of their own and host genes, regulating fundamental cellular processes in immunity, apoptosis, and key steps in the herpesvirus life cycle such as latency, lytic infection and the transition from latent to lytic infection [10], [11], [12], [13]. MiRNAs have been found to be expressed by many herpesviruses examined so far [14], [15], [16], [17], [18]. Recently, five viral miRNAs were found in PRV-infected porcine dendritic cells (DC) [19].

Recent advances in high throughput sequencing technologies have greatly increased the speed and sensitivity of miRNA discovery. While northern blot hybridization is an acceptable method for confirming the expression of miRNAs, it suffers from a low sensitivity. Stem-loop RT-PCR is a simple and highly sensitive approach to specifically identify mature miRNAs based on known unique sequences [20], [21]. Therefore, combined strategies are needed to discover novel miRNAs for more effectively and convincingly.

Compared to other herpesvirus-encoded miRNAs in lytic or latent infection [18], [22], [23], [24], the reported PRV-encoded miRNAs in DC are limited. We hypothesized that PRV infection in epithelial cell line should generate more miRNAs to breakthrough the first line of physical defense. In this study, we made a comprehensive analysis on the PRV miRNA expression profiles utilizing combined strategies mentioned above. Our interests were to determine whether PRV encodes more than the 5 miRNAs identified so far and to characterize the PRV miRNAome in porcine epithelial cell line. We also analyzed both host and viral targets of PRV miRNAs with the aim to identify the functions of these viral miRNAs. The results revealed the complexity of PRV miRNA expression profile and interaction network formed by viral miRNAs and their targets.

## Results

### Analysis of Small RNA by Deep Sequencing

**Figure 1:**
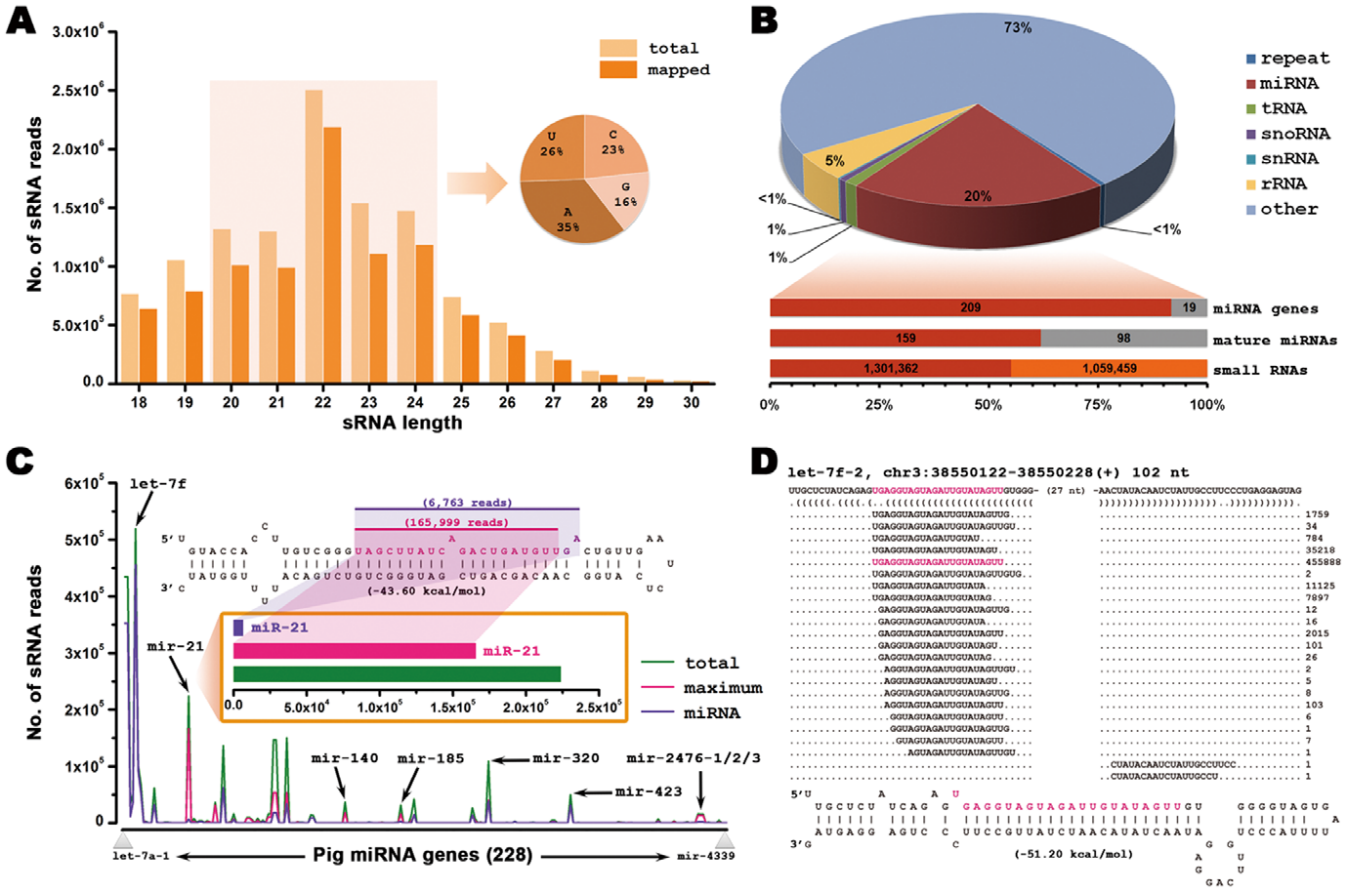
Characteristics of total small RNAs. (A) Length distribution of total sRNAs (718,321 sequences, corresponding to 11,744,709 reads) in pseudorabies virus (PRV)-infected PK-15 cells. sRNAs between 20-nt and 24-nt in length are highlighted by light yellow background and their first nucleotide distribution is shown in pie chart. (B) Pie chart summarizing the different classes of sRNAs. Some sRNAs can be mapped to more than one category. To make every unique sRNA mapped to only one category, the following priority rule was used: microRNA (miRNA)>repeat>rRNA>tRNA>snoRNA>snRNA. The annotated miRNAs from miRBase database (Release 17) were shown in detail with bars below: the number of miRNA genes (top) supported by at least one sRNA read (red) and not generated any sRNA (gray); the number of mature miRNAs (middle) detected in the library (red) and not detected (gray); the number of sRNAs (bottom) derived from mature miRNA loci (red) and other loci (orange) from the miRNA genes. (C) The total reads (green), maximum reads (pink) and annotated mature miRNA reads (purple) from each known miRNA gene. Chart was plotted in ascending order of miRNA identifiers (leftmost, let-7a-1; rightmost, miR-4339). Several miRNAs (i.e., let-7f, miR-21, miR-140, miR-185, miR-320, miR-423, miR-2476-1, miR-2476-2 and miR-2476-3) are indicated with arrows. miR-21 was shown in detail in the above chart. Top panel: the predicted secondary structure of miR-21 with the annotated mature miRNA (purple) from miRBase database and a novel miRNA isoform (pink) detected from our study. Bottom panel: the expression level of the annotated miRNA and its isoform. (D) A novel miRNA (let-7f-2) identified from the swine genome. Top: the predicted secondary structure of let-7f-2; bottom: the distribution of sRNA reads across its hairpin. More newly indentified miRNAs are listed in Table S3.

After natural route of infection through oronasal cavity, PRV firstly replicates in the nasal epithelium cells, and then it enters free nerve endings of trigeminal as well as other sympathetic, parasympathetic, and facial nerve neurons innervating peripheral mucosa [25]. To investigate the miRNA profiles in infected epithelial cell line, PK-15 cells were infected with PRV Ea strain as described in Materials and Methods, and small RNAs (sRNAs) were isolated and analyzed by deep sequencing (Solexa/Illumina technology). This resulted in 11,744,709 reads between 18-nt and 30-nt in length, representing 718,321 distinct sequences remained for further analysis, and 9,281,056 (79%) reads were perfectly matched either to the pig genome sequence (Sscrofa9.2) or to the PRV genome (GenBank accession NO. NC_006151). About 70% sRNAs range from 20-nt to 24-nt in length, most of which with 5′ A or 5′ U (Figure 1A). Of the total reads, 20% were mapped to the miRNA hairpin stuctures, and 7% mapped to the annotated non-coding RNA genes or repeat sequences (Figure 1B).

### Analysis of host miRNA expression profile

Previously, Anselmo et al. were able to detect 156 known and 27 new porcine miRNAs in PRV infected dendritic cells [10]. In this study, 209 host miRNA genes (92% of 228 miRBase-annotated genes in Release 17) generated at least one sRNA sequence from their precursors. However, only 159 (62%) mature miRNAs were detected, which accounted for 55% (1,301,362/2,360,821) of all reads mapped on their precursors (Figure 1B). These data indicate that other new mature miRNA isoforms (isomiRs) were detected from the sequencing library. In fact, we found that 62 (27%) miRNA precursors produced more abundant sRNA reads (>100 reads) than the previously annotated miRNAs (Figure 1C and Table S1). For example, miR-21 is not the predominant sRNA (with 6,763 reads) in the miR-21 precursor, while another sRNA, which is 2-nt shorter than miR-21 at the 3′ end, is of the most abundance. Besides, we identified 39 novel host miRNAs in the sequencing library (Table S2). One such newly identified miRNA, named let-7f-2 (a member of let-7f family), is shown in Figure 1D.

### Identification of PRV-encoded miRNAs from deep sequencing data

**Figure 2:**
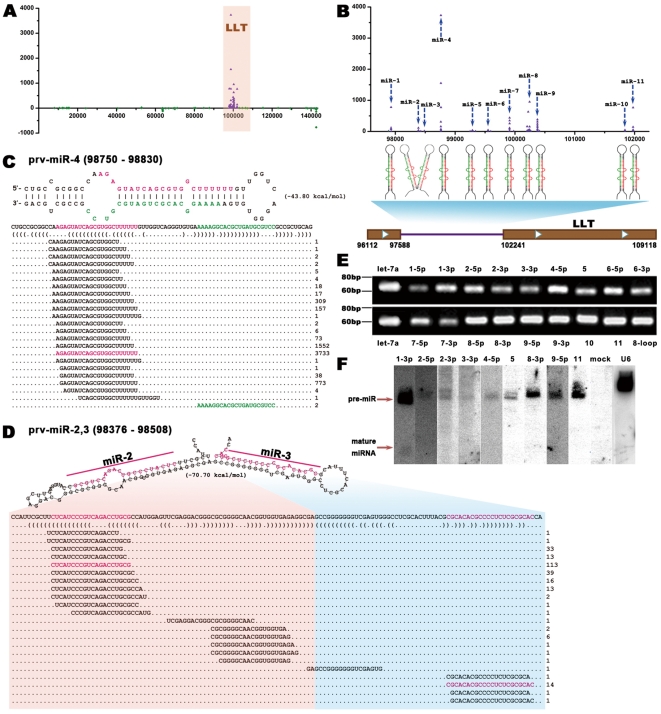
MicroRNAs derived from the Large Latency Transcript (LLT) of PRV. (A) The distribution of small RNA (sRNA) derived from the PRV genome. The genomic region of LLT is highlighted in pink background, and sRNAs are dotted in LLT and terminal region. (B) 11 miRNAs (named as prv-miR1 to 11) derived from the LLT intron. The expression patterns of the miRNAs are plotted in the top and the corresponding schematic structures were shown in the middle, and boundaries of the LLT gene are indicated at the bottom. For both A and B, x-axis indicates the genomic coordinates of the PRV genome and y-axis the read count of each sRNA. sRNAs generated from the sense strand are shown above the x-axis and anti-sense strand below. Only sRNAs with more than three reads in the library were plotted after alignment by the 5′ terminus. (C) The predicted secondary structure of prv-miR-4 (top) and the distribution of sRNA reads across its hairpin (bottom). (D) prv-miR-2 and 3 produced from the jointed hairpin precursor. The predicted secondary structure was shown at the top and the alignment of sRNA reads at the bottom. For both C and D, the number of reads mapped to the miRNA precursors is indicated in the right side. Mature miRNAs and miRNA*s are denoted in red and green, respectively, and numbers in parentheses indicate the genomic positions of miRNA precursors. (E) Stem-loop RT-PCR identification of 17 mature miRNAs and a loop-derived small RNA originated from prv-miR-1 to prv-miR-11. (F) Northern blot analysis of selected PRV miRNAs. Twenty micrograms of total RNA for each miRNA were separated on a 15% denaturing polyacrilamide gel, transfered and hybridized using miRNA special radiolabeled probes. U6 snRNA served as loading control and PK-15 mock-infected cells as a blank control.

In total, 5 966 distinct sRNA sequences from 22 179 individual reads were mapped to the PRV genome, and the majority of them were located in the region of LLT (Figure 2A). Following the alignment of sequences to the hairpin-like structures on the genome, 11 unique miRNA genes at 13 loci were identified. These viral miRNAs were designated as prv-miR-1 to prv-miR-11 (Figure 2B and Table 1). Notably, prv-miR-10 and prv-miR-11 are each present in two copies in the PRV genome, and those located in the intron of LLT were designated as prv-miR-10-1 and prv-miR-11-1, while the homologous miRNAs in the terminal repeat region (TR) were named as prv-miR-10-2 and prv-miR-11-2. All 11 LLV intronic miRNA genes are interspersed and arranged in the same transcriptional orientation. Some of these pre-miRNAs encode mature miRNAs both at 5′ arm and 3′ arm. For instance, prv-miR-4, the most abundant pre-miRNA, encodes AGAGUAUCAGCGUGGCUUUUUU at 5′ arm, and AAAAGGCACGCUGAUGCGUCC at 3′ arm (Figure 2C and Table 1). This led to the recovery of 19 mature miRNAs originated from the 11 pre-miRNAs (Table 1) and 7 novel miRNA precursors, namely prv-miR-2, -3, -4, -7, -8, 10, and -11, comparing with previous study [10]. Interestingly, prv-miR-2 and prv-miR-3 share an overlapping region in their precursors, and presumably they are produced from the same conjoined precursor (Figure 2D).

**Table 1:** Summary of results of the experimental validation of PRV encoded miRNAs.

miRNA	Sequence(5′–3′)	Length	Strand and Position	Reads	Experimental Confirmation		
					Stem-loop PCR	Northern Blot	
						Pre	Mature
Prv-miR-1-5′	GACGGCUCCUGGGGCUGAAAGC	22	+97887–97908	26	+	ND	ND
Prv-miR-1-3′	UCUCACCCCUGGGUCCGUCGC	21	+97930–97950	779	+	+	+
Prv-miR-2-5′	CUCAUCCCGUCAGACCUGCG	20	+98386–98405	113	+	+	−
Prv-miR-2-3′	CGCGGGGCAACGGUGGUGAG	20	+98426–98445	6	+	+	−
Prv-miR-3-5′	GAGCCGGGGGGGUCGAGUG	19	+98450–98468	1	−	ND	ND
Prv-miR-3-3′	CGCACACGCCCCUCUCGCGCAC	22	+98485–98506	14	+	+	−
Prv-miR-4-5′	AGAGUAUCAGCGUGGCUUUUUU	22	+98762–98783	3733	+	+	−
Prv-miR-4-3′	AAAAGGCACGCUGAUGCGUCC	21	+98800–98820	2	−	ND	ND
Prv-miR-5	UGAGUGGAUGGAUGGAGGCGAG	22	+99910–99931	439	+	+	−
Prv-miR-6-5′	CGUACCGACCCGCCUACCAGG	21	+99550–99570	44	+	ND	ND
Prv-miR-6-3′	CUUGGCAGCGGGUGGGUACC	20	+99595–99614	4	+	ND	ND
Prv-miR-7-5′	CCGGGGGGUUGAUGGGGAU	19	+99288–99306	36	+	ND	ND
Prv-miR-7-3′	ACCACCGUCCCCCUGUCCCU	20	+99342–99361	6	+	ND	ND
Prv-miR-8-5′	GUGGGGGCGAAGAUUGGGUU	20	+100221–100240	631	+	+	−
Prv-miR-8-3′	CAACCCUUCUGGAGCCCUACC	21	+100267–100287	8	+	+	−
Prv-miR-9-5′	AUCGAGGAGAUGUGGAGGGG	20	+100375–100394	382	+	+	−
Prv-miR-9-3′	CCCUCCCCCGCAUCUCUUCUC	21	+100411–100431	12	+	ND	ND
Prv-miR-10-1	CCGAGCCUGCCCCUUCCGUCGCA	23	+101838–101860	46	+	ND	ND
Prv-miR-10-2			−142764–142742				
Prv-miR-11-1	AGGCUGGGAGUGGGGACGGAAGA	23	+101975–101997	768	+	+	−
Prv-miR-11-2			−142627–142605				
Prv-miR-8-loop	UGAGAGACUAGAACCGGUGUUCU	23	+100245–100267	951	+	ND	ND

The sequences and positions of PRV encoded miRNAs are correlated with Genbank accession no. BK001744. miRNAs are shown as either detected (+), not detected (−) or not done (ND).

### Detection of PRV-encoded miRNAs by stem-loop RT-PCR and northern blot analysis

To confirm the deep sequencing data, stem-loop RT-PCR (see Materials and Methods) was adopted to verify the expression of mature PRV miRNAs. Host miRNA let-7a and 17 mature viral miRNAs, except for prv-miR-3-5′ and prv-miR-4-3′, were detected using this approach. The amplified fragments were approximately 65-nt long as expected (Figure 2E, Table 1). To compare the PRV miRNA expression profile in other cell types, another pig kidney cell line IB-RS-2 and a bovine kidney cell line MDBK were infected, respectively, and subjected to viral miRNA detecting using the same method. All of these miRNAs and the loop-derived small RNA were detected except miR-1-5′ in PRV-infected IB-RS-2 cells (Figure S1). While in the bovine kidney cells, the detected viral miRNA number is limited to 5. Interestingly, the loop-derived small RNA was also detected in MDBK cells (Figure S1). Viral miRNAs displaying higher expression levels were selected for northern blot analysis. The pre-miRNAs of prv-miR-4, prv-miR-2 and prv-miR-3 corresponding to Figure 2C and Figure 2D were detected (Figure 2F and Table 1). The blot pattern shows one band for prv-miR-2-5′, but two bands for prv-miR-2-3′, indicating that prv-miR-2 and prv-miR-3 might be generated by different mechanisms. Four other pre-miRNAs (prv-miR-1, prv-miR-5, prv-miR-9 and prv-miR-11) and one mature miRNA, prv-miR-1-3′, were detected by northern blot as well (Figure 2F and Table 1).

### Characterization of PRV miRNAs

Of the above 17 detected viral miRNAs (Table 1), 4 miRNAs share the conserved but not completely identical sequences with those previously reported PRV miRNAs in DC, i.e., prv-miR-P1-3′ versus prv-miR-1, prv-miR-P5 versus prv-miR-3, prv-miR-P6-5′ versus prv-miR-2, and prv-miR-P9-5′ versus prv-miR-5 [10]. These conserved viral miRNAs from infected DC and PK-15 are isomiRs which exhibit high sequence identity but do not share consistent terminal sequences, suggesting a different splicing site preferences between DC and epithelial cells.

**Figure 3:**
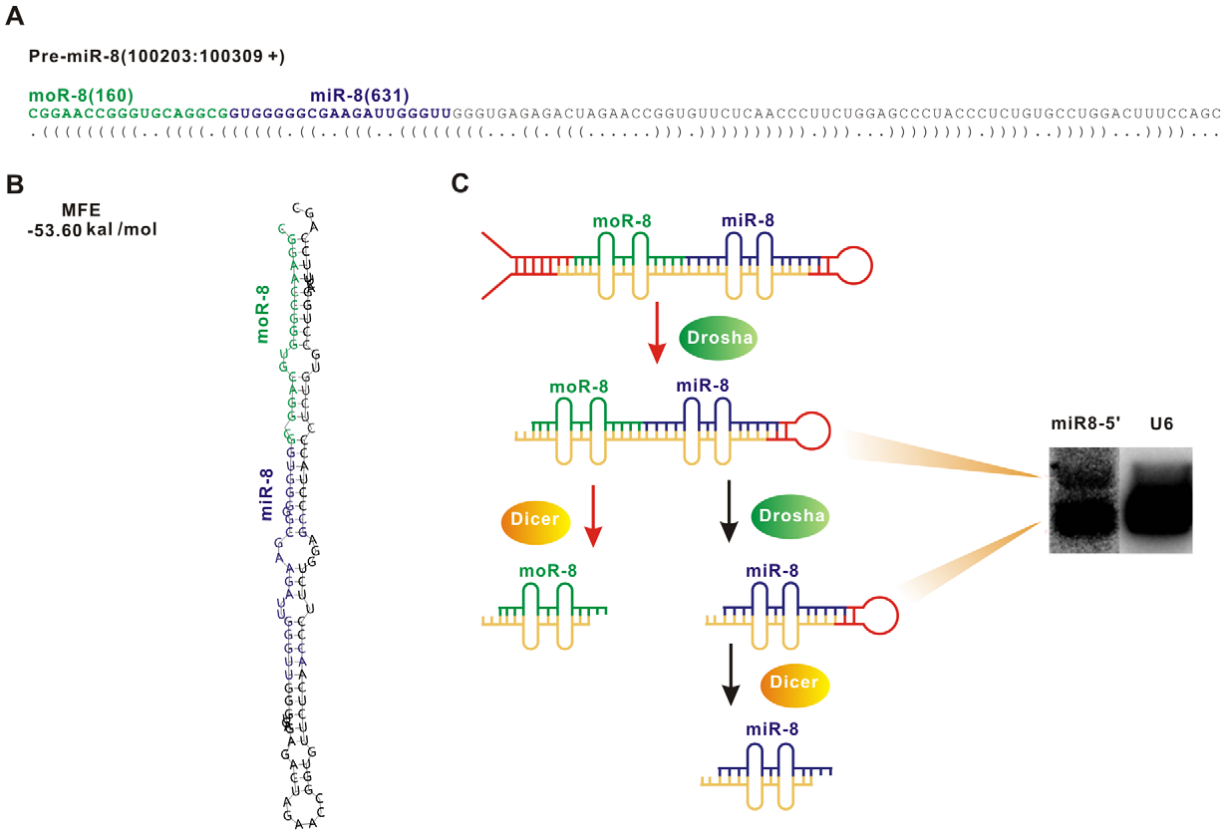
Model for Processing of pre-miR-8 in PK-15 and DC. (A) Sequence and pair condition of pre-miR-8. Sequence and relative locations of moR-8 and miR-8 are also shown. (B) Sequence and secondary structures of pre-miR-8. Shown are locations of moR-8 (purple) and miR-8 (green). (C) Model for processing of pre-miR-8 is shown. Red arrows represent the moR-8 processing in DC and all arrows represent the processing of miR-8 precursors in PK-15. Northern blot analyse (right) was performed to examine the processing products in PK-15 cell, with different bands showing miR-8 precursors.

Strikingly, the 18-nt small RNA previously annotated as prv-miR-P4 is actually a miRNA offset RNA (moRNA) [26] of miR-8-5p originated from prv-miR-8. Thus, we designate this RNA as moR-8 in the subsequent assays. Of note, the previous study detected moR-8 with an abundance of 450 in DC but failed to recover mature miR-8. However, in our study we recovered mature miR-8 with a reads of 631 and moR-8 with 160 counts (Figure 3A). Based on the differential expression of miR-8 and moR-8 between in epithelial cells and in DC, we proposed a biogenesis model of processing mechanism of pri-miR-8 (Figure 3B and 3C). i) In both DC and PK cells, pre-prv-miR-8 is cleaved by Drosha, ii) in DC cells, the moR8-miR8 miRNA is cleaved once by Dicer to generate moR-8, while iii) After that, in PK cells, the moR8-miR8 precursor is cleaved by Dicer to generate moR-8 or by Drosha to generate miR-8 hairpin which is further cleaved by Dicer to generate the mature mature miR-8. While in DC, the moR8-miR8 precursor is cleaved by only Dicer to generate moR-8.

**Figure 4:**
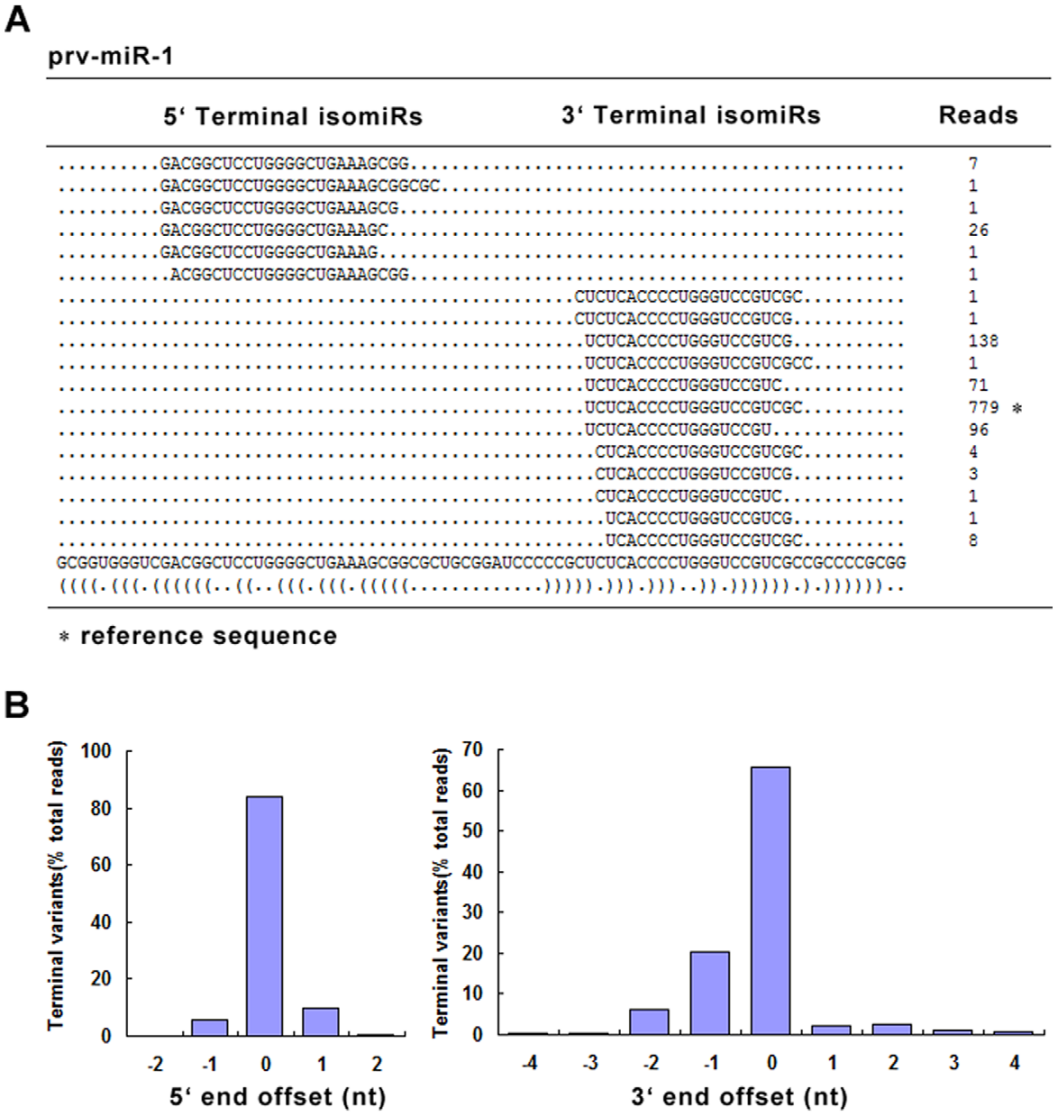
Sequence variation in PRV miRNAs recovered from deep sequencing of PK-15 cells. (A) An example of terminal sequence variation of PRV miRNA prv-miR-1. Shown are the distribution and corresponding sequence reads of isomiRs. (B) Distribution of 5′ ends (left) and 3′ ends heterogeneity (right) of PRV miRNAs. For each miRNA, the most abundant isomiR is regarded as corresponding reference sequence and designated as nucleotide position 0. Extensive nucleotides at 5′ ends are numbered negative and nucleotides at 3′ numbered positive.

Additional analysis of the small RNA sequences which mapped on pre-miRNAs revealed another interesting phenomenon. The majority of the PRV mature miRNAs show end heterogeneity in both 5′ ends and 3′ends, as previously observed for Rhesus lymphocryptovirus (rLCV) [24], Epstein-Barr virus (EBV) [27], Kaposi's sarcoma-associated herpesvirus (KSHV) [23]. However, these miRNAs exhibit fewer sequence variations at the 5′ ends, while variations at the 3′ ends are fairly common (Figure 4). In subsequent text, we thus consider the most abundant of all the isomiRs of a given set mapping to the same pre-miRNA as the “reference” mature miRNA”.

Unexpectedly, following the alignment of the sequences to the hairpin-like structures of PRV genome, a 23-nt small RNA with 951 reads located at the end loop of prv-miR-8 was recovered. This end-loop-derived sRNA is more abundant than the miRNAs from 3′ arm with 8 reads and from 5′ arm of the stem with 631 reads (Table S2). This small RNA could also be detected by stem-loop RT-PCR (Figure 2E and Table 1).

In conclusion, it is reasonable that PRV, as well as other alphaherpeviruses, encode miRNAs with different polymorphism and tend to use DNA/RNA to their maximum potential in different cell types.

### Self-regulation by viral miRNAs

**Figure 5:**
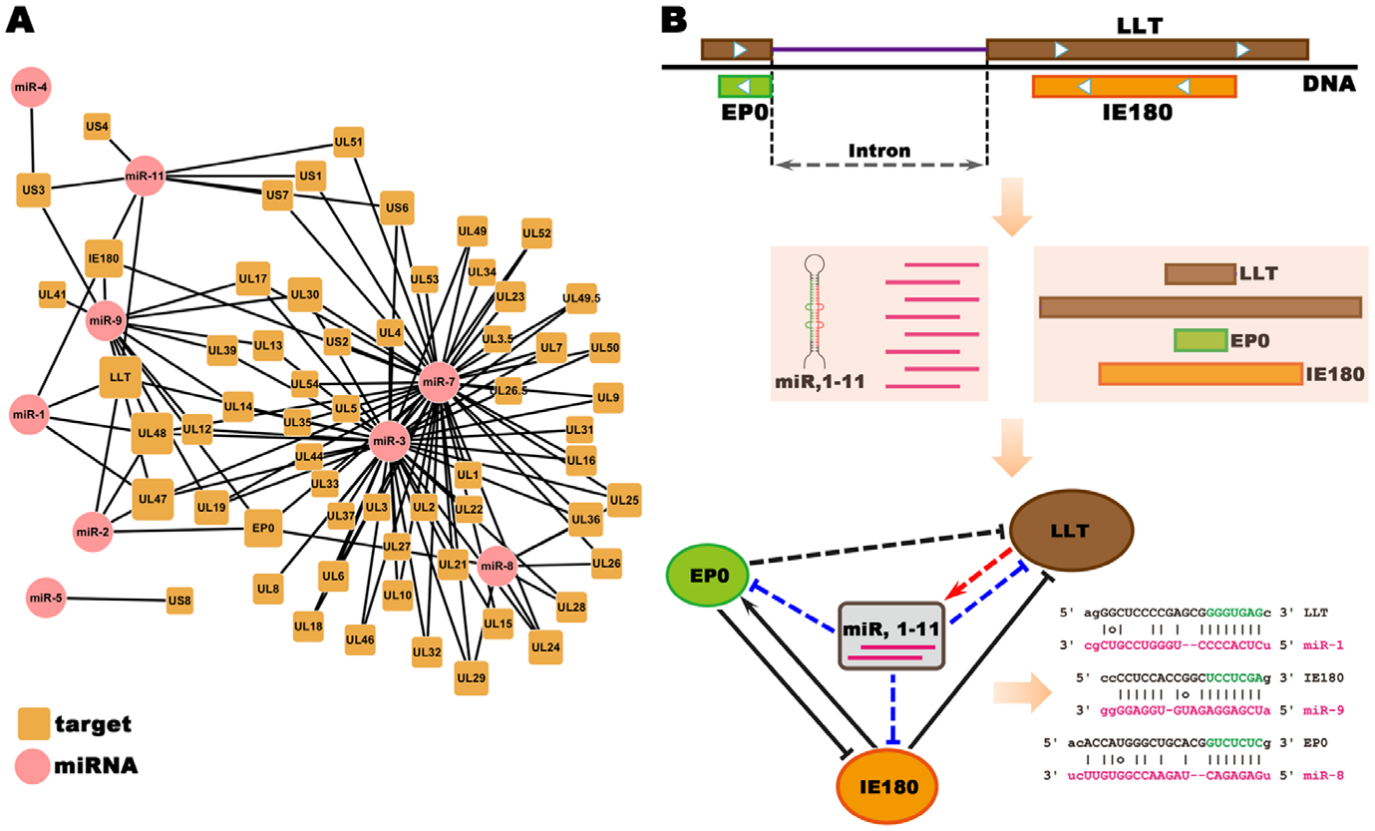
Gene regulatory network formed by viral microRNAs and their target genes in PRV genome. (A) A predicted network between the eleven identified miRNAs (round rectangles) and their putative target genes (circles) in the PRV genome. (B) A self-regulatory sub-network formed by intronic miRNAs and their host genes at the LLT gene locus. LLT gene contains one intron, and EP0 (Early Protein 0) and IE180 (Immediate Early Protein 180) are transcribed from its anti-sense strand (top). Eleven miRNAs are generated from the intronic region of the LLT gene (middle). Possible targets for the 11 miRNAs include the LLT itself, EP0 and IE180 (bottom left). Solid line indicates regulatory relationship verified by previous studies and dashed line mentioned by this study. Several miRNA target sites are displayed at the bottom right.

Viral miRNAs could have a regulatory impact on PRV transcripts to support the virus replication cycle and allow it to escape the host-immune system [28]. We scanned the 70 PRV-encoded genes, including LLT, with the miRNA sequences to uncover possible sequence homologies. To predict putative PRV targets for the 11 identified viral miRNAs, relatively stringent criteria were utilized to reduce fasle-positive interactions. Briefly, putative target genes were firstly identified by miRanda program with parameters: -sc 130 -en 30, and strict alignments (G:U wobble is not allowed) [29], and then filtered with several additional restrictions including high local AU composition, positioning away from the centers of long UTRs and expanded base pairing within miRNA nucleotides 13–16 [30] (see Materials and Methods). Results show that 60 genes are targeted by one or more miRNAs. Some miRNAs (like prv-miR-1 and prv-miR-3) can target multiple genes, while some genes (like LLT, IE180 and EP0) are targeted by multiple miRNAs. These viral miRNAs and target genes form a complex regulatory network (Figure 5A). IE180 is the only immediate early gene of PRV, EP0 is a crucial early regulatory gene, and LLT is a structural RNA that is highly associated with the latency of PRV. EP0 and IE180 are antiparallel to and overlap the two exons of LLT. Interestingly, LLT itself and the two important *trans* regulators, IE180 and EP0, which in return regulate the transcription of LLT [31], [32], were predicted to be targeted by several miRNAs (Figure 5A). Given the fact that these miRNAs are derived from the intron region of LLT, a miRNA-mediated feedback loop is proposed, where the miRNAs are placed in the core of the sub-network (Figure 5B).

### Regulatory impact of viral miRNAs on host genes

**Figure 6:**
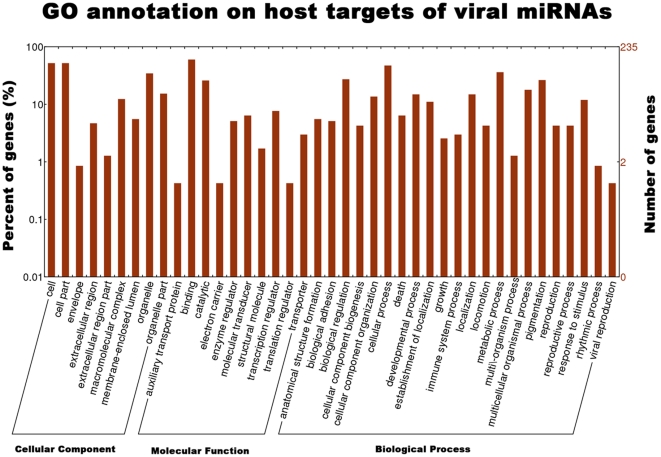
GO annotation on host target genes of viral miRNAs. GO functional analysis utilizing WEGO show that 235 targets belong to several kinds of functional genes involved in immune process, viral reproduction, metabolic process and cell death, indicating the regulatory potential of these miRNAs in virus-host interaction.

MiRNA-regulated pathways were reported to be linked to many respects of host-virus interaction in which viral miRNAs have a regulatory effect on cellular transcripts in order to exert their functions, e.g. transcription supressor gene *BACH1* downregulated by KSHV miR-K12-11, antiproliferative and anti-angiogenic gene *THBS1* inhibited by KSHV miR-K12-6-3p, antiviral factor *MICB* downregulated by HCMV miR-UL112-1, pro-apoptotic protein *PUMA* downregulated by EBV miR-BART5, and interferon-inducible T-cell chemoattractant *CXCL11* downregulated by EBV miR-BHRF1-3 [11]. Using similar criteria mentioned above (see Materials and Methods), 235 host genes were predicted to be target by the 11 viral miRNAs (Table S4). In order to investigate biological roles of these viral miRNAs, GO annotation was performed for the putative host target genes. Results revealed that these target genes were involved in immune system process, viral reproduction, cell metabolic process and others (Figure 6). Furthermore, GO enrichment analysis showed that these target genes were functionally enriched in apoptosis, cell death, and negative regulation of binding (*P*<0.05, Table S5). These results indicate that viral miRNAs play important regulatory roles in the virus-host interaction.

## Discussion

PRV has been considered as a useful model for the investigation of herpesvirus biology by particularly of viral life cycle and host-virus interactions [33]. Even though numerous reports have documented that many protein-coding genes contribute to PRV pathogenesis, the detailed mechanisms underlying the acute infection, latency, and transition from latency to reactivation are not fully understood. Recent studies revealed that miRNAs play important roles in virus life cycle [10]. Five miRNAs were reported in PRV-infected DC, however, an accurate functional analysis of PRV miRNAs is unlikely to be achieved without the discovery of the PRV miRNAome in other cell types, especially epithelial cells and neuronal cells. To our knowledge, this study is the first to elucidate the miRNA expression profiles in PRV-infected epithelial cells. Our data identified 11 PRV-encoded miRNA genes with 17 mature miRNAs, and confirmed the expression of known porcine miRNA genes with mature isomiRs and novel miRNAs in the infected PK-15 cells. In addition, we made an elaborate analysis of PRV miRNA transcriptome, revealed the polymorphisms of these viral miRNAs, provided insight into the complex regulatory network of PRV miRNAs and their viral and host target genes.

Virus can affect cellular miRNA expression profile to make a replication niche. For example, miR-17/92 cluster are prominently downregulated by HIV [34]. In another study, miR-100 and miR-101, both target rapamycin (PI3K/Atk/mTOR) pathway, are strongly downregulated by HCMV [35]. Viral miRNAs also influence host miRNA expression by competing to use the same miRNA processing system [24]. In our study, 209 out of the 228 miRBase-annotated miRNA genes were detected in host cells infected by PRV. Only 159 mature miRNAs reads were mapped perfectly on their precursors, but 62 highly abundant mature isomiRs were also detected. In addition, 39 novel host miRNA genes were expressed in PK-15 cells. The highly abundant presence of isomiRs and some other miRNA expression pattern of PK-15 cells may be associated with PRV infection.

There is no obvious latency stage of PRV in PK-15 cells. However, the detection of various miRNAs originated from PRV LLT in PK-15 cells indicates robust transcription from this region during lytic infection. Our data showed that the LLT intron (∼4.6-kb long) encodes a cluster of 11 miRNA genes with at least 17 mature miRNAs from the same strand, and thus we think that the PRV LLT intron functions as a primary miRNA precursor. The rare non-protein-coding region of viral genome acts as a miRNA-coding sequence indicates that the genome is densely filled with both coding and non-coding RNAs. Among the 11 miRNAs identified in our study, only 4 of them were previously reported in PRV-infected DC, while the other 7 are novel. Anselmo et al. [19] provided the first report about PRV encoded miRNAs. It is known that miRNA expression depends on different tissues and different development stages [9]. The epithelial cell line we chose are different with the dendritic cells used by Anselmo [19]; Besides, we collected infected PK-15 cells from seven timepoints, while Anselmo [19] collected infected dendritic cells at a single timepoint (8 hpi). This might be the reason why more abundant viral miRNAs were detected in epithelial cells than in infected dendritic cells.

Prv-miR-10 and prv-miR-11 are located in the repeat regions; therefore they both have two reverse copies. In herpesviridae, some elements such as BICP0 and the replication origin in bovine herpesvirus, have two copies due to their location in the repeat regions. This redundancy of miRNAs seems to be common in herpesviruses and important to virus normal life cycle. The significance of this redundancy is still unknown.

MiRNAs recognize mRNA highly dependent on full sequence complementarity of seed sequences between the miRNAs and the their target mRNAs, located between nucleotides 2 to 8 from the 5′ end of the miRNA [36]. Thus, terminal stability of miRNA 5′ end is crucial for target discrimination. Our study show that the presence of terminal variations of PRV miRNAs are not only at the 5′ ends but also more predominantly at the 3′ ends. It is possible that these isomiRs regulate a distinct but overlapping set of mRNA targets *in vivo*, reflecting a highly efficient and complex regulatory mechanism of PRV miRNAs. The biogenesis of moRNAs is not as clear compared to that of miRNAs, and whether moRNAs are loaded into RISC to play some regulatory roles is currently unknown [26]. Recent studies indicated that moRNAs coexist with and are expressed at far lower frequencies than related miRNAs [23], [37], [38], [39]. Our result showed that moRNAs were expressed in epithelial cell line and had an abundant expression level. Interestingly, our study first reported an overlapping structure in virus-encoded miRNAs. The remarkable difference of expression level and northern blot pattern indicated a special mechanism for PRV's miRNA processing.

Normally, the sliced top loop is degraded by relevant nuclease [40]. But in our study, a 23-nt small RNA derived from top loop of miR-8 appeared with high frequency. The sequencing data show that this small RNA is more abundant than the 5′ and the 3′ mature miRNAs. By using stem-loop RT-PCR, the loop-derived small RNA was further confirmed to exist. The abundance and length similarity suggest that this small RNA might have a special function in PRV life cycle.

Several studies have demonstrated the downregulation of cellular and/or virus mRNAs by herpesvirus miRNAs [41]. It is surprising that these viral miRNAs are putatively targeting a majority of PRV genes even after extremely strict sequence screening in our study. In addition, they have the potential to recognize numerous host genes which play roles in immune system process, viral reproduction, cell metabolic process. A recent study reported that intronic small RNAs produced by plants mediate DNA methylation of host genes [42]. In our study, prv-miR-1, prv-miR-3 and prv-miR-11 were predicted to target both LORF1 and LORF2, while prv-miR-2 and prv-miR-9 target LORF1, which reveal that there exist a similar self-regulation mechanism of PRV LLT and its miRNAs. Usually miRNAs do not inhibit transcription but rather inhibit translation of their targets. It thus seems that the binding of viral miRNAs and their primary transcripts will not influence the expression level of the miRNAs themselves. Further study is needed to clarify the self-regulation mechanism of LLT and its miRNAs. The PRV EP0 gene exhibits inhibitory activity for IE180 [43]. IE180 inhibits LLT promoter LAP1 in neuronal cells during latent infection [44], [45]. Since IE180, EP0 and LLT are clustered in the genome [3], these genes could reciprocally be regulated by an antisense mechanism like the same in herpes simplex virus type 1 (HSV-1) [46], [47], [48]. Our result indicates that they could also be regulated by miRNA parthway. This kind of regulation might be vital to the virus's latency and reactivation.

A previous study demonstrated that the US3-null PRV still inhibit the apoptosis of neurocyte but induces strong pro-apoptosis in epithelial cells [49]. However, the molecular mechanism of this phenomenon is unclear. The analysis of the host targets indicate that the viral miRNAs can target genes related to apoptosis and cell death. Besides, a large proportion of these targets belong to metabolic process and immune system process.

Taken together, our study unveils the PRV miRNA expression profiles, especially the detailed and distinct viral miRNAs in PRV-infected epithial cells. We consider that the PRV miRNAs constitute at least a distinct structural subset of the PRV genome, fulfilling an important but as yet to be discovered functional role in the virus life cycle. We believe these data provide important clues to and accelerate the understanding of the pathogenesis of PRV and other herpesviruses, as well as the host processing pathways of miRNAs of both cells and viruses.

## Materials and Methods

### Virus and cells

PRV Ea, a virulent wild type strain [50], was utilized in this study. PK-15, IB-RS-2 and MDBK cell lines, purchased from China Center for Type Culture Collection (Wuhan), were cultured at 37°C in Dulbecco's modified Eagle's medium supplemented with 10% heat-inactivated fetal bovine serum. Cells were passaged into cell dishes to approximately 90% confluency, and infected with PRV of 10 PFU per cell. Cells were harvested at 2, 4, 6, 9, 12, 15, and 18 hours post infection (hpi) and resuspended in Trizol (Invitrogen).

### RNA isolation and sequencing

Total RNAs from PRV-infected PK-15 cells at the above time points were extracted according to the manufacturer's instructions, electrophoresed in formaldehyde-denatured agarose gel, and quantified with spectrophotometer (NanoDrop 2000). 10 µg RNA extracted from each timepoint was mixed and then subjected to BGI (Huada Genomics Institute Co. Ltd, China) for solexa sequencing of small RNAs. The same mixed RNA sample was used in the subsequent experiments including stem-loop RT-PCR and northern blot analysis.

### Stem-loop RT-PCR

The stem-loop RT-PCR was carried out as previously described [21], [51]. Briefly, aliquot of 20 µg of RNA mixture was treated with DNase I (Promega), then cDNAs were synthesized using superscript III transcriptase (Invitrogen) together with miRNA-specific stem-loop RT primers (Table S6). Next, PCR was performed for each miRNA using the transcription product and miRNA-specific forward primer and universal reverse primer (Table S6), and cellular miRNA let-7a was used as positive control. The amplification condition was as follows: 95°C 5 min, followed by 40 cycles of 95°C 20 s, 60°C 20 s and 72°C 20 s, and subsequent elongation at 72°C for 5 min.

### Northern blot

For PRV miRNAs detection by northern blot analysis, 20 µg of RNA mixture for each miRNA was mixed with RNA loading buffer (Ambion) and electrophoresed in a denaturing 15% polyacrylamide gel containing 8 M urea. The gel was run for 20 min prior to RNA loading, and followed by 2 h electrophoresis at 100 V after loading. Then the separated RNA was transferred from gel to Hybond N^+^ nylon membrane (GE) for 8 min at 20 v using an Bio-Rad semi-drying instrument, and cross-linked using UV light. DNA probes with sequences complementary to miRNAs (Table S7) were end-labeled with [γ-^32^P]ATP. Membranes were pre-hybridized with PerfectHybTM Hybridization Solution (TOYOBO) for 1 h at 42°C, and then hybridized to radiolabeled probes overnight at the same temperature. The hybridized blots were washed three times in 2×SSC buffer with 0.1% SDS at 42°C for 15 min, and then exposed to phosphor screen for 24 h except for U6 (used as a uniform loading control for all northern blots) for 30 min, with mock-infected cells as negative control. Finally, signals were detected using a cyclone storage phosphor system (Fujifilm, FLA 5100).

### Data sources

PRV full genome sequence was retrieved from the NCBI Genome database (ftp://ftp.ncbi.nih.gov/refseq/release/viral/). Pig (Sus scrofa) genomic sequence, gene annotation information and repeat sequences were retrieved from the UCSC Genome Browser (http://genome.ucsc.edu/cgi-bin/hgGateway?org=Pig&db=susScr2, SGSC Sscrofa9.2/susScr2), and the annotated pig miRNAs were from miRBase (http://www.miRbase.org/, Release 17). The known noncoding RNAs (only consider rRNA, tRNA, snRNA and snoRNA) were obtained from the Rfam database (http://rfam.janelia.org/; release 9.1).

### Bioinformatics analysis of small RNAs

The small RNA sequencing reads produced by the Illumina Genome Analyzer were subjected to the following process: (1) filter and remove out low quality reads, (2) trim three prime adaptor sequences, (3) remove adaptor contaminations formed by adaptor ligation, and (4) retain only trimmed reads of sizes from 18 to 30 nt long.

The filtered short reads were then mapped to the Rfam database, pig repeat database, and the pre-miRNA sequences from miRBase, respectively, using the Bowtie algorithm [52]. After excluding those sRNAs screened by the above categories, the remaining short reads were subjected to novel miRNA prediction using “MIREAP” program (http://sourceforge.net/projects/miReap/).

### MiRNA target prediction and GO analysis

MiRNA targets were predicted by miRanda algorithm [53] with parameters: -sc 130 -en 30, and strict alignments (G:U wobble is not allowed) were required in the seed region. The predicted targets were then filtered by several additional features of site context proposed by Grimson et al [30], including high local AU composition near the site, positioning away from the centers of long UTRs and expanded base pairing within miRNA nucleotides 13–16 (G:U wobble is allowed). The filtered targets were then subjected to GO functional analysis utilizing WEGO program [54] to create histograms of GO annotation against cell component, biological process and molecular function.

### Gene enrichment analysis

GO enrichment analysis of the target genes was performed using GO::TermFinder [55] to detect the significantly enriched GO terms of the target gene sets (235 genes total) compared to the genome-wide background. Hypergeometric test and Benjamini & Hochberg false discovery rate (FDR) were performed using the default parameters to adjust the P-value (P<0.05).

## Supporting Information

Figure S1 **Stem-loop RT-PCR identification of PRV-encoded miRNAs and the loop-derived small RNA in IB-RS-2 and MDBK.** IB-RS-2 and MDBK cells were mock infected or infected with PRV as described in Materials and Methods. Following the RNA extraction, stem-loop RT-PCR was carried out. The PCR products were detected in 3% agarose gel. The upper two pictures show PRV miRNA expression profile in mock- and PRV-infected MDBK cells, while the lower two show PRV miRNA expression profile in mock- and PRV-infected IB-RS-2 cells.
(TIF)

Table S1 **Summary of the expression data for known detected porcine miRNAs.** “MIR gene” represents the miRNA genes annotated in miRBase, Release 17.0. “Total reads” represents total small RNA reads mapped to the precursors of miRNA genes. “Maximum reads” and “Sequences of maximum reads” represent the number of reads and their sequences of the most abundant small RNAs corresponding to the miRNA genes. “Total miRNA reads” represents the total reads of the mature miRNAs. “miRNA 1”, “miRNA 2” and “miRNA sequence” represent the mature miRNAs, their sequences and reads. Note that some miRNA genes produce more than one mature miRNAs, which are denoted as 1, 2, etc. The background is colored according to the total number of small RNA reads of the miRNA genes.
(XLS)

Table S2 **Terminal heterogeneity of PRV-encoded miRNAs.** Small RNA sequences and their coresponding reads mapped on the precursors of 11 viral miRNA genes. The sequences of the miRNA genes and their secondary structures (shown in dot-bracket notation) predicted by RNAfold program (Hofacker 2003) are shown (middle in each section). Small RNAs mapped to the sense strand of miRNA genes are listed above the predicted structure and antisense strand below. Mature RNAs are shown in red and relevant miRNA* are shown in green. The loop-derived small RNA of miR-8 is shown in purple.
(XLS)

Table S3 **Novel porcine miRNAs expressed in PK-15 cells.** Shown are sequences mapped on the corresponding precursors and chromosomes (yellow). The sequences of the miRNA genes and their secondary structures (shown in dot-bracket notation) predicted by RNAfold program (Hofacker 2003) are shown (middle in each section). Small RNAs mapped to the sense strand of miRNA genes are listed above the predicted structure and antisense strand below. Mature sequence and sequence reads (red) of novel swine miRNAs predicted with “MIREAP” program.
(XLS)

Table S4 **Predicted swine target genes by PRV miRNAs.** 235 target genes were rocoverd using miRanda and filtered as previously described by Grimson et al [22]. Shown are miRNAs and corresponding target gene IDs.
(XLS)

Table S5 **GO term enrichment analysis of swine target genes.** Total 235 predicted target genes were performed by GO enrichment analysis using GO::TermFinder (P<0.05).
(XLS)

Table S6 **Primers used for stem-loop RT-PCR for detecting mature PRV miRNAs.**
(XLS)

Table S7 **Probes used for northern blot analysis of selected PRV miRNAs.**
(XLS)
