# Supplementary figures and images for: Pseudorabies Virus Infected Porcine Epithelial Cell Line Generates a Diverse Set of Host MicroRNAs and a Special Cluster of Viral MicroRNAs
Source: PLoS One. 2012 Jan 23;7(1):e30988. doi: 10.1371/journal.pone.0030988 (PMC3264653; doi:10.1371/journal.pone.0030988)

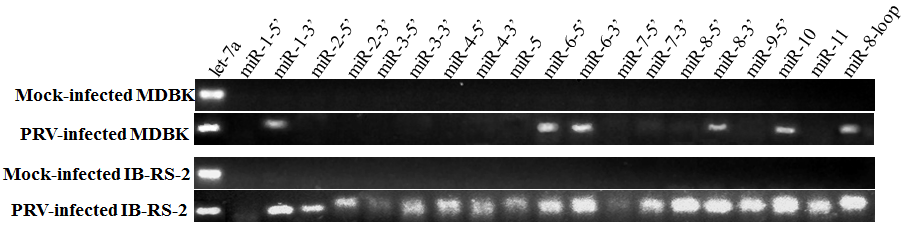

Supplement: Figure S1 — Stem-loop RT-PCR identification of PRV-encoded miRNAs and the loop-derived small RNA in IB-RS-2 and MDBK. IB-RS-2 and MDBK cells were mock infected or infected with PRV as described in Materials and Methods. Following the RNA extraction, stem-loop RT-PCR was carried out. The PCR products were detected in 3% agarose gel. The upper two pictures show PRV miRNA expression profile in mock- and PRV-infected MDBK cells, while the lower two show PRV miRNA expression profile in mock- and PRV-infected IB-RS-2 cells. (TIF) [file pone.0030988.s001.tif]
